# MicroRNA profiles following metformin treatment in a mouse model of non-alcoholic steatohepatitis

**DOI:** 10.3892/ijmm.2015.2092

**Published:** 2015-02-06

**Authors:** AKIKO KATSURA, ASAHIRO MORISHITA, HISAKAZU IWAMA, JOJI TANI, TEPPEI SAKAMOTO, MIWA TATSUTA, YUKA TOYOTA, KOJI FUJITA, KIYOHITO KATO, EMIKO MAEDA, TAKAKO NOMURA, HISAAKI MIYOSHI, HIROHITO YONEYAMA, TAKASHI HIMOTO, SHINTARO FUJIWARA, HIDEKI KOBARA, HIROHITO MORI, TOSHIRO NIKI, MASAFUMI ONO, MITSUOMI HIRASHIMA, TSUTOMU MASAKI

**Affiliations:** 1 Department of Gastroenterology and Neurology, Kagawa University School of Medicine, Kita-gun, Kagawa, Japan; 2 Life Science Research Center, Kagawa University School of Medicine, Kita-gun, Kagawa, Japan; 3 Department of Immunology and Immunopathology, Kagawa University School of Medicine, Kita-gun, Kagawa, Japan; 4 Department of Gastroenterology and Hepatology, Kochi Medical School, Koichi, Japan

**Keywords:** MCD, hepatic steatosis, liver fibrosis

## Abstract

Non-alcoholic steatohepatitis (NASH) is one of the most common causes of chronic liver disease and is considered to be a causative factor of cryptogenic cirrhosis and hepatocellular carcinoma. microRNAs (miRNAs) are small non-coding RNAs that negatively regulate messenger RNA (mRNA). Recently, it was demonstrated that the aberrant expression of certain miRNAs plays a pivotal role in liver disease. The aim of the present study was to evaluate changes in miRNA profiles associated with metformin treatment in a NASH model. Eight-week-old male mice were fed a methionine- and choline-deficient (MCD) diet alone or with 0.08% metformin for 15 weeks. Metformin significantly downregulated the level of plasma transaminases and attenuated hepatic steatosis and liver fibrosis. The expression of miRNA-376a, miRNA-127, miRNA-34a, miRNA-300 and miRNA-342-3p was enhanced among the 71 upregulated miRNAs, and the expression of miRNA-122, miRNA-194, miRNA-101b and miRNA-705 was decreased among 60 downregulated miRNAs in the liver of MCD-fed mice when compared with control mice. Of note, miRNA profiles were altered following treatment with metformin in MCD-fed mice. miRNA-376a, miRNA-127, miRNA-34a, miRNA-300 and miRNA-342-3p were down-regulated, but miRNA-122, miRNA-194, miRNA-101b and miRNA-705 were significantly upregulated in MCD-fed mice treated with metformin. miRNA profiles were altered in MCD-fed mice and metformin attenuated this effect on miRNA expression. Therefore, miRNA profiles are a potential tool that may be utilized to clarify the mechanism behind the metformin-induced improvement of hepatic steatosis and liver fibrosis. Furthermore, identification of targetable miRNAs may be used as a novel therapy in human NASH.

## Introduction

Non-alcoholic steatohepatitis (NASH) is a non-alcoholic fatty liver disease (NAFLD), and patients who have a fatty liver develop inflammation and fibrosis (1). Ten to 20% of NAFLD patients can progressively develop NASH, liver fibrosis, cirrhosis and hepatocellular carcinoma (HCC) (1,2). The natural progression from NAFLD to NASH remains unknown, and the reason for certain NAFLD patients developing steatohepatitis and cirrhosis remains to be elucidated. However, Day and James (3) proposed a ‘two-hit’ model that suggests a second hit is needed to develop NASH.

Although simple hepatic steatosis is the result of the accumulation of various lipids (4) and a benign process in the majority of patients, NASH may reflect different disease entities. Notably, NASH patients with less steatosis first exhibit inflammation (2). In addition, inhibition of hepatic tissue necrotic factor a (TNFα) improves steatosis in *ob/ob* mice (5,6), and decreasing the expression of interleukin-10 (IL-10) derived from Kupffer cells can improve hepatic steatosis (7). Lipid accumulation may be caused by a stress response that is induced by the inflammation of hepatocytes. Tilg and Moschen (1) reported that inflammatory mediators derived from various tissues, especially from the gut and adipose tissue, may play a central role in the cascade of inflammation and fibrosis.

Metformin was introduced into clinical practice as an oral biguanide drug for the treatment of type 2 diabetes in the 1950s (8). It inhibits glucose production in the liver and improves hyperglycemia. Findings of several recent reports showed that metformin has various effects on non-alcoholic steatohepatitis (9,10). Metformin prevented and reversed steatosis and inflammation caused by NASH without affecting peripheral insulin resistance (10). However, the mechanism underlying this improvement of steatosis, inflammation and fibrosis remains unknown.

miRNAs are non-coding RNAs that are 20–25 nucleotides in length, and they have been shown to negatively regulate mRNA expression in animals, plants and viruses (11). In general, miRNAs bind to the 3′-untranslated region of protein coding genes and inhibit gene expression (12). Findings of previous studies have identified a critical role for miRNA in human NASH (13,14) and HCC (15). These data suggest that miRNAs have the potential use as a therapeutic target for preventing disease progression and prognosis in NASH patients. However, the miRNA profiles following metformin treatment in a mouse model of NASH remain unclear.

In the present study, we analyzed the miRNA expression pattern to elucidate the mechanism of action and efficacy of metformin using an experimental non-diabetic model without affecting peripheral insulin resistance.

## Materials and methods

### Chemicals

Metformin (metformin hydrochloride) was purchased from Wako Pure Chemical Industries, Ltd., Tokyo, Japan.

### Animal model and experimental design

Eight-week-old male C57BL/6N mice were purchased from CLEA Japan Inc. Mice were housed for 15 weeks on a 12-h light/dark cycle, and food and water were accessible *ad libitum*. Mice were fed either a methionine- and choline-deficient (MCD) diet (Oriental Yeast, Tokyo, Japan) or a normal diet. Mice were divided into three experimental groups and fed for 15 weeks. Group 1 was given a methionine- and choline-deficient (MCD) diet (MCD, n=10). Group 2 was fed an MCD diet with 2.4 mg/day metformin (Wako Pure Chemical Industries) (MCD + metformin, 2.4 mg/day, n=10). Group 3 was fed normal chow (NC, n=7). Group 2 was fed with an MCD diet and treated with 2.4 mg/day metformin given in the drinking water. The dose of metformin was calculated at 2.4 mg/mouse/day and corresponds to 4,800 mg/60 kg in a human. Group 3 was fed a standard diet and received untreated drinking water *ad libitum*. The mice were fed for 15 weeks to recreate the advanced stages of steatohepatitis. After 15 weeks on each diet, the mice were euthanized and the liver and body weight were measured. Livers were fixed in 10% formalin or flash frozen in liquid nitrogen for histological analysis. The samples were stored at −80°C until further analysis. All animal procedures were performed in accordance with the guidelines of the Committee on Experimental Animals of Kagawa University, Kagawa, Japan.

### Blood sampling and analysis

Blood samples were obtained from the right ventricle, and the levels of AST, ALT and ALP were measured by an autoanalyzer (TBA-200FR NEO; Toshiba Medical Systems Corp., Tokyo, Japan).

### Histological evaluation and immunohistochemistry

To determine whether metformin decreased MCD-induced steatosis and fibrosis in the liver, Oil red-O and Azan staining was performed, respectively. In all the experimental groups, 5-*μ*m sections of formalin-fixed and paraffin-embedded liver samples were processed for Azan staining. Oil red-O staining was also performed with all the liver samples to estimate the degree of hepatic steatosis. Areas of the digital photomicrographs were quantified with a computerized image analysis system (ImageJ).

### Analysis of miRNA microarrays

The total RNA of each liver sample was extracted using a miRNeasy Mini Kit (Qiagen, Tokyo, Japan) according to the manufacturer’s instructions. Total RNA was measured using an RNA 6000 Nano kit (Agilent Technologies, Tokyo, Japan). The samples were labeled using a miRCURY Hy3/Hy5 Power Labeling kit and were hybridized on a mouse miRNA Oligo chip (v. 17.0; Toray Industries, Inc., Tokyo, Japan). Scanning was conducted with a 3D-Gene Scanner 3000 (Toray Industries). 3D-Gene extraction version 1.2 software (Toray Industries) was used to read the raw intensity of the image. To determine the change in miRNA expression between the metformin-treated and control samples, the raw data were analyzed via GeneSpring GX v10.0 (Agilent Technologies). The samples were first normalized relative to the 28S RNA level and then baseline-corrected to the median of all the samples.

Replicate data were consolidated into two groups, including those from metformin-treated animals and those from control animals. The data were organized using the hierarchical clustering and analysis of variance (ANOVA) functions in the GeneSpring software. Hierarchical clustering was completed using the clustering function (condition tree) and a Euclidean correlation as a distance metric. A two-way ANOVA and asymptotic P-value computation without any error correction was performed on the samples to search for the miRNAs that varied most prominently across the different groups. The P-value cut-off was set to 0.05. Only changes >50% for at least one of the time-points within each sample were considered significant. All the analyzed data were scaled by global normalization. The statistical significance of differentially expressed miRNAs was analyzed by the Student’s t-test. Our microarray data in the present study were submitted as a complete data set to the NCBI Gene Expression Omnibus (GEO) no.: GSE55593; control vs. MCD (http:ncbi.nlm.nih.gov/=GSE55593) and no.: GSE55523; MCD vs. MCD + metformin (http:ncbi.nlm.nih.gov/=GSE55523).

### Heatmap

To show alterations in the expression level of nine miRNAs, we created a heatmap in which each cell represents the expression level of each miRNA for four individual subjects from the MCD-fed mice that were treated with and without metformin. The heatmap was color-coded according to a log 2-transformed expression level. The center level of the color code was set as the median value for all the values used to create the heatmap. Thus, white was considered the mean number; red, an increase and blue, a decrease in the heat map.

### Statistical analysis

Data are shown as the means ± standard deviation (SD). Statistical significance was defined as P<0.05 for the unpaired t-test and as P<0.025 for the Bonferroni corrections.

## Results

### Metformin attenuates the development of MCD-induced NASH

**Figure 1:**
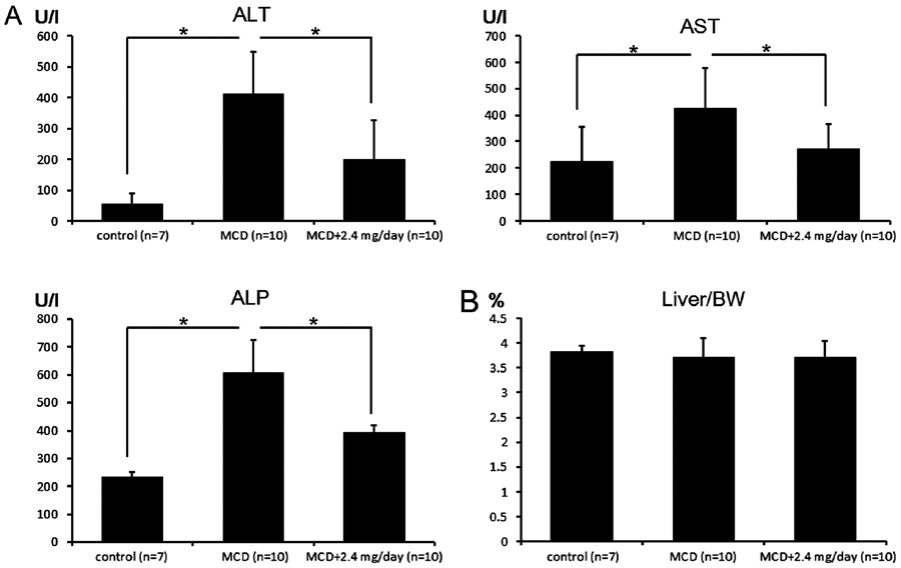
(A) The effect of metformin on ALT, AST and ALP levels in MCD-fed mice. MCD-fed mice had higher levels of plasma ALT, AST and ALP, while metformin prevented this increase. Data are shown as the mean ± SD (^*^P<0.025 when compared with the normal group or MCD-fed mice). (B) The effect of metformin on the liver/body weight ratio of MCD-fed mice. There was no significant difference among the groups. Data are shown as the mean ± SD.

**Figure 2:**
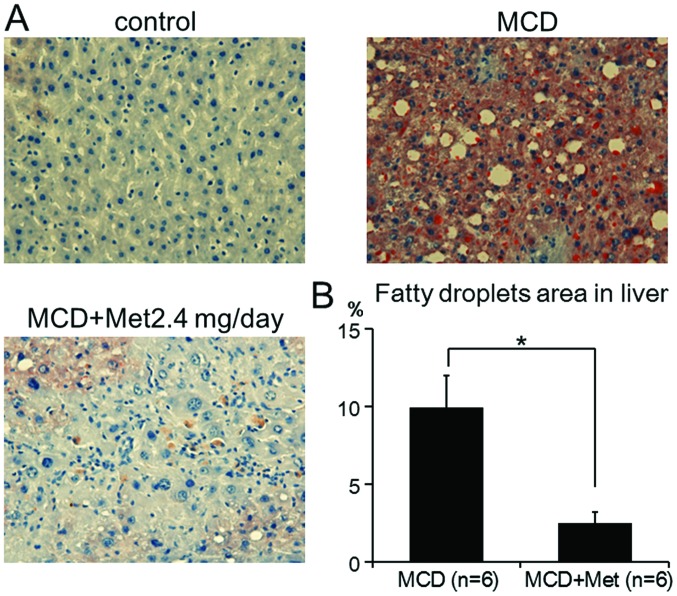
Metformin attenuates the development of MCD-induced hepatic steatosis. (A) Paraffin-embedded sections were stained with Oil red-O (original magnification, x200). Livers of the MCD-fed mice exhibited severe steatosis. Metformin attenuated the development of MCD-induced hepatic steatosis. (B) Image analysis of the Oil red-O-stained liver sections was performed using an image analysis system. Metformin significantly attenuated hepatic steatosis. Data are shown as the mean ± SD (^*^P<0.05).

To determine the effect of metformin on the development of NASH, mice were fed an MCD or normal diet. In MCD-fed mice, the levels of plasma ALT, AST and ALP were higher than those for mice fed a normal diet. Treatment of MCD-fed mice with metformin significantly attenuated this increase (P<0.025; significant differences were confirmed with a Bonferroni correction) (Fig. 1A). In the present study, the liver/body weight ratio was not significantly different between MCD-fed mice treated with and without metformin (Fig. 1B). To determine whether metformin decreased MCD-induced liver steatosis, Oil red-O staining was completed. Consistent with the results from the plasma ALT, AST and ALP levels, MCD-fed mice treated with 2.4 mg metformin showed significantly suppressed development of MCD-induced liver steatosis by 75% (9.91±2.03 vs. 2.51±0.70%, P<0.001) (Fig. 2). These results suggested that metformin suppressed the inflammation and steatosis induced by MCD.

### Liver fibrosis is suppressed by metformin

**Figure 3:**
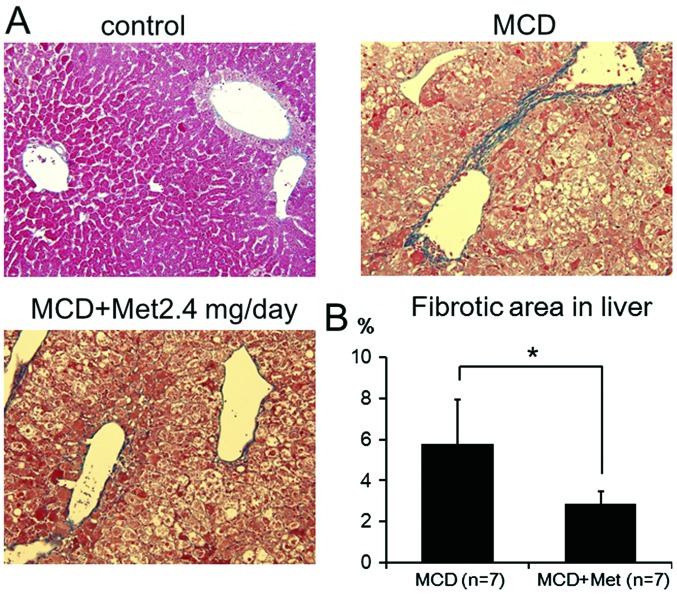
Metformin prevents liver fibrosis. (A) Paraffin-embedded sections were stained with Azan staining (original magnification, x200). (B) Image analysis of the Azan-stained liver sections was performed using an image analysis system. Metformin significantly attenuated liver fibrosis. Data are shown as the mean ± SD (^*^P<0.05).

Histological analysis of the liver was performed to determine whether the liver fibrosis induced in MCD-fed mice was inhibited by metformin. Perivenular fibrosis was detected around the central vein in MCD-fed mice. By contrast, metformin treatment significantly downregulated the extent of perivenular fibrosis (Fig. 3).

### miRNA expression in the liver tissue of MCD-fed mice

**Figure 4:**
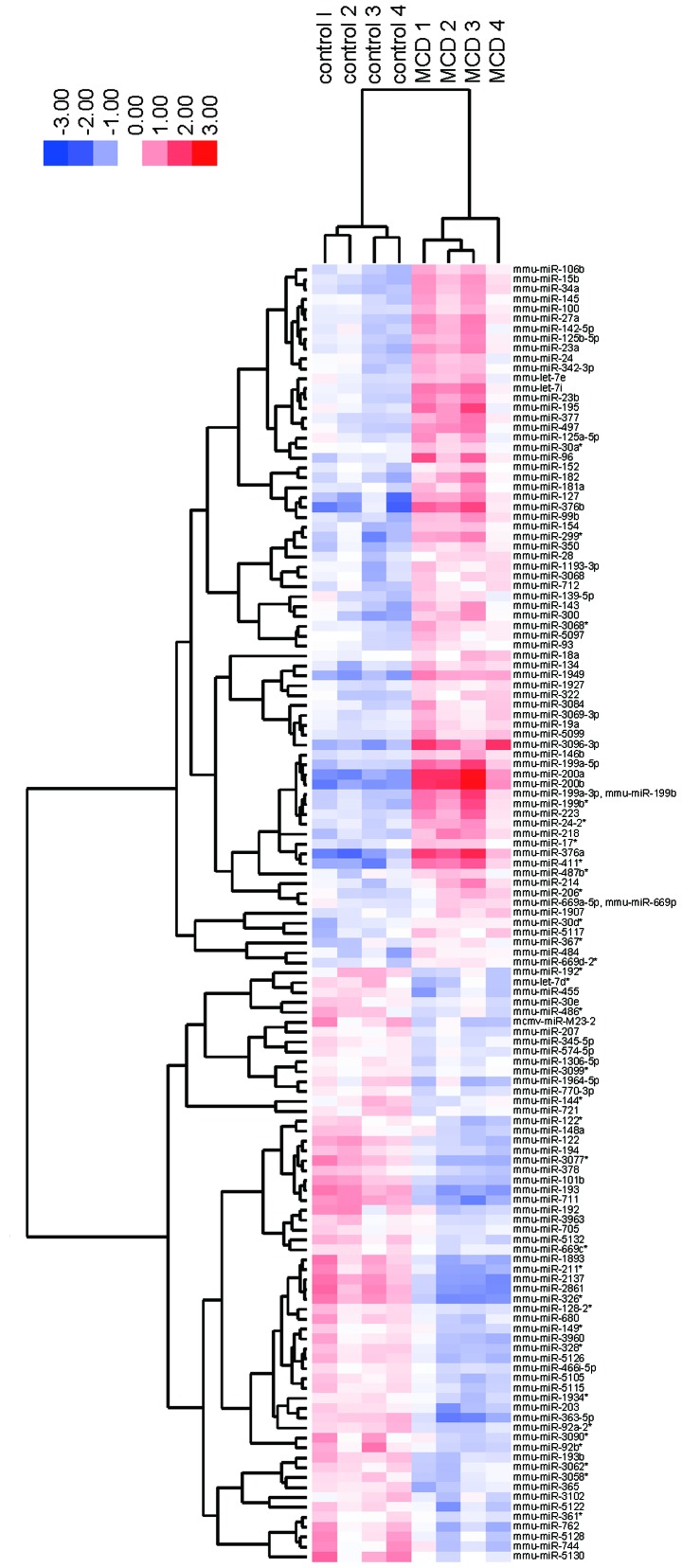
Hierarchical clustering of liver tissues from MCD-fed mice and control group. Liver tissue was clustered according to the expression profiles of 131 differentially expressed miRNAs between the MCD-fed mice and the control group. The analyzed samples are reported in columns and the miRNAs are presented in rows. The miRNA clustering tree is shown on the left, and the sample clustering tree appears at the top. The color scale shown at the top indicates the relative expression level of miRNAs, with red representing a high expression level and blue, a low expression level.

To elucidate the miRNA profiles during the development of NASH, we analyzed the expression levels of 1,135 mouse miRNA probes using liver tissue from control and MCD-fed mice. As shown in Table IA, 71 miRNAs were significantly upregulated, and 60 miRNAs were downregulated in MCD-fed mice. Unsupervised hierarchical clustering analysis using a Pearson’s correlation showed that MCD-fed mice clustered separately from the control group (Fig. 4). These subsets of 131 miRNAs exhibited significant alterations in their expression levels between the MCD-fed and control mice.

### Differences in miRNA expression in liver tissue from mice treated with metformin

**Figure 5:**
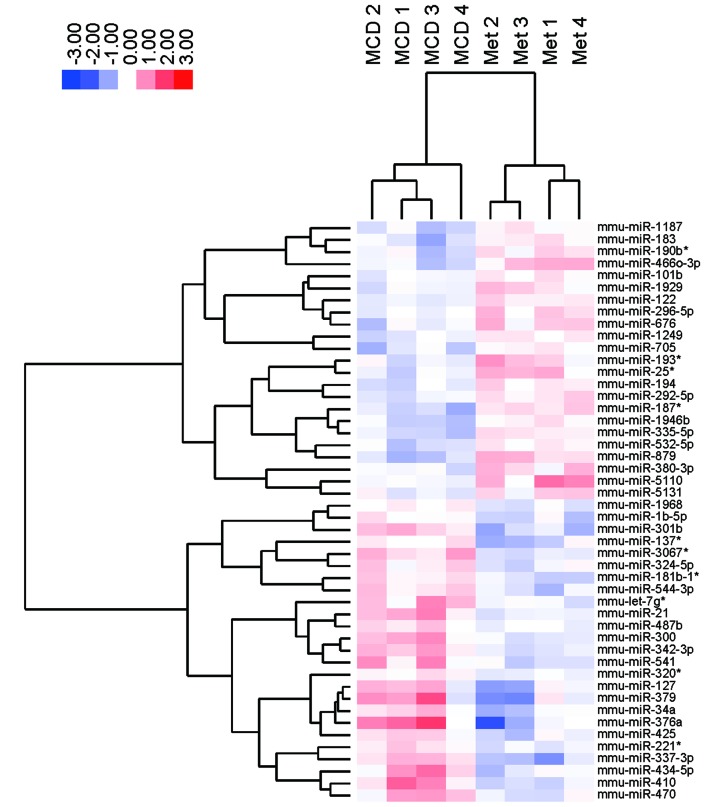
Hierarchical clustering of liver tissues from MCD-fed mice with or without metformin. Liver tissue was clustered according to the expression profiles of 48 differentially expressed miRNAs between metformin-treated and non-treated MCD-fed mice.

**Figure 6:**
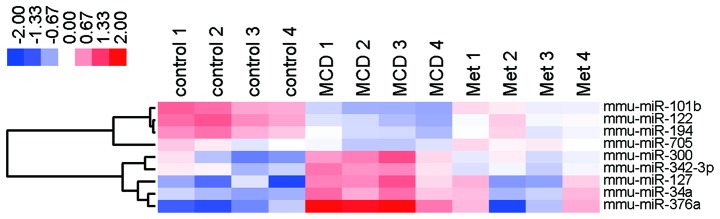
Metformin regulates the microRNA expression induced by an MCD diet. miR-376a, miR-127, miR-34a, miR-300 and miR-342-3p were downregulated, and miR-122, miR-101b, miR-194 and miR-705 were upregulated in MCD-fed mice after treatment with metformin.

miRNA profiles were examined in MCD-fed mice after metformin treatment. As shown in Table IB, 23 miRNAs were significantly upregulated, and 25 miRNAs were downregulated in MCD-fed mice treated with metformin. Unsupervised hierarchical clustering analysis using a Pearson’s correlation showed that MCD-fed mice without metformin clustered separately from the MCD-fed mice treated with metformin (Fig. 5). These subsets of 48 miRNAs exhibited significant alterations in their expression levels between the MCD-fed mice with or without metformin. Notably, miR-122, miR-194, miRNA-101b, and miRNA-705 were upregulated and miRNA-376a, miRNA-127, miRNA-34a, miRNA-300 and miRNA-342-3p were downregulated in the liver tissue of MCD-fed mice treated with or without metformin (Table IB and Fig. 6). The four upregulated miRNAs, i.e., miR-122, miR-194, miRNA-101b and miRNA-705, in mice treated with or without metformin were consistent with four of the 60 downregulated miRNAs from the control group and MCD-fed mice. The five downregulated miRNAs i.e., miRNA-376a, miRNA-127, miRNA-34a, miRNA-300 and miRNA-342-3p, were identical to five of the 71 upregulated miRNAs in control and MCD-fed mice.

## Discussion

Most cases of NAFLD remain free of inflammation, with only 10–20% of these patients developing inflammation and fibrosis (1). Therefore, NASH is thought to be the progressive form of NAFLD. Various hits, such as endoplasmic reticulum stress, adipocytokines, and innate immunity derived from the gut and/or the adipose tissue, may promote liver inflammation (1). Lin *et al* (6) reported that metformin was effective at reversing fatty liver disease most likely via the reduced production of tumor necrosis factor in hepatocytes. Additionally, Kita *et al* (10) demonstrated that metformin can prevent and reverse the development of steatosis and inflammation in the liver of a NASH dietary mouse model. However, the mechanism of metformin-induced inhibition of NASH development remains unclear. To elucidate the relationship between miRNA and NASH development, miRNA profiles were determined following metformin treatment in a non-diabetic mouse model of nonalcoholic steatohepatitis.

Of a number of upregulated miRNAs, miRNA-376a, miR-127, miR-34a, miR-300, miR-342-3p were downregulated following metformin treatment in MCD-fed mice. Recently, miR-376a downregulation has been shown to be associated with arsenic trioxide (ATO)-induced apoptosis in human retinoblastoma cells (16). In addition, the downregulation of miR-127 facilitates hepatocyte regeneration after partial hepatectomy (17). Furthermore, miR-34a, which directly targets sirtuin 1 (SIRT1), were inhibited by ursodeoxycholic acid (UDCA) in the rat liver and activated by disease severity in human NASH (18). Taken together, it is suggested that metformin suppresses steatosis, inflammation and fibrosis in the liver of MCD-fed mice through metformin-induced down-regulation of these miRNAs.

By contrast, miRNA-122 and miRNA-194 were significantly upregulated by metformin out of the nine miRNAs that were downregulated in the NASH liver of MCD-fed mice. Hu *et al* (14) recently reported that miRNA-122 is a liver-specific miRNA and acts as a suppressor of cell proliferation and carcinogenesis in hepatocytes (19). Additionally, it has been described that miRNA-122 is downregulated in NASH and may alter lipid metabolism in the liver (13). Furthermore, Tsai *et al* (20) have shown that loss of miRNA-122a induces steatosis, fibrosis and hepatocarcinogenesis. Currently, several target genes of miRNA-122 have been shown to be involved in hepatocarcinogenesis, such as a distintegrin and metalloproteinase family 10 (ADAM10), serum response factor (SRF) (21), insulin-like growth factor 1 receptor (Igf1R) (22), cyclin G1 (23) and Wnt1 (24). However, the target gene of miRNA-122 involved in lipid metabolism remains elusive (25). Similar to miRNA-122, downregulation of miRNA-194 enhances the expression of frizzled-6 (FZD6) and promotes tumorigenesis in the adult liver (26). miRNA-194 is also considered to be a marker of hepatic epithelial cells and inhibits the metastasis of liver cancer cells (27). In various types of cancer, such as gastric (28), endometrial cancer (29,30), renal cell carcinoma (31) and colorectal cancer (32) miRNA-194 inhibits tumor invasion and metastasis. Taken together, it is suggested that one of the downstream targets of the metformin-induced pathway is miRNA-122 and/or miRNA-194.

In conclusion, we identified nine key miRNAs that were modulated by metformin in the NASH liver of MCD-fed mice. Our findings also suggest that the regulation of these key miRNAs serves as a novel therapeutic candidate for human NASH.

## Figures and Tables

**Table I:** Statistical analysis of miRNAs in liver tissues between MCD-fed mice and control group (P<0.05) and between MCD-fed mice treated and untreated with metformin (P<0.05).

A, Statistical analysis of miRNAs in liver tissues between MCD-fed mice and control group		
Upregulated microRNA	P-value	Fold (MCD/control) mean ± SD
mmu-miR-200b	0.00016	10.454±5.110
mmu-miR-200a	0.00021	9.572±3.666
mmu-miR-376a^a^	0.00062	8.165±3.935
mmu-miR-3096-3p	0.00043	5.578±1.768
mmu-miR-376b	0.00367	5.326±1.933
mmu-miR-411*	0.00126	4.670±2.692
mmu-miR-1949	0.00003	3.677±0.672
mmu-miR-199a-5p	0.00152	3.646±1.482
mmu-miR-199a-3p, -199b	0.00374	3.460±1.875
mmu-miR-199b*	0.00387	3.193±1.448
mmu-miR-127^a^	0.00488	3.042±0.584
mmu-miR-299*	0.00749	2.959±1.861
mmu-miR-96	0.02105	2.797±1.802
mmu-miR-195	0.04001	2.536±1.569
mmu-miR-300^a^	0.00971	2.487±1.277
mmu-let-7i	0.00635	2.478±0.882
mmu-miR-223	0.00614	2.374±1.042
mmu-miR-218	0.00168	2.365±0.392
mmu-miR-497	0.01038	2.347±0.865
mmu-miR-15b	0.00131	2.329±0.517
mmu-miR-23a	0.00458	2.326±0.666
mmu-miR-27a	0.00415	2.304±0.770
mmu-miR-34a^a^	0.00054	2.300±0.519
mmu-miR-182	0.01445	2.288±0.883
mmu-miR-377	0.00406	2.273±0.853
mmu-miR-23b	0.01032	2.187±0.787
mmu-miR-99b	0.00218	2.124±0.326
mmu-miR-142-5p	0.02372	2.077±0.971
mmu-miR-24-2*	0.00260	2.013±0.649
mmu-miR-143	0.02913	1.999±0.763
mmu-miR-214	0.02618	1.981±1.168
mmu-miR-125b-5p	0.00692	1.973±0.646
mmu-miR-106b	0.00414	1.913±0.432
mmu-miR-350	0.00433	1.877±0.612
mmu-miR-154	0.00706	1.867±0.504
mmu-miR-134	0.00576	1.822±0.308
mmu-miR-5099	0.00408	1.776±0.397
mmu-miR-145	0.01834	1.766±0.446
mmu-miR-342-3p^a^	0.01310	1.726±0.445
mmu-miR-322	0.00698	1.725±0.393
mmu-miR-206*	0.01010	1.718±0.550
mmu-miR-146b	0.00117	1.713±0.362
mmu-miR-125a-5p	0.04886	1.681±0.555
mmu-miR-100	0.00402	1.653±0.355
mmu-miR-5117	0.02651	1.645±0.628
mmu-miR-3084	0.01816	1.644±0.322
mmu-miR-3068*	0.00874	1.624±0.221
mmu-miR-3068	0.03368	1.616±0.607
mmu-miR-24	0.04066	1.610±0.371
mmu-miR-181a	0.02427	1.610±0.241
mmu-miR-1193-3p	0.03269	1.600±0.340
mmu-miR-484	0.04737	1.594±0.474
mmu-miR-712	0.01563	1.592±0.346
mmu-miR-17*	0.00911	1.589±0.423
mmu-let-7e	0.04746	1.546±0.483
mmu-miR-19a	0.00447	1.517±0.233
mmu-miR-3069-3p	0.00558	1.511±0.143
mmu-miR-28	0.01445	1.498±0.392
mmu-miR-487b*	0.04660	1.482±0.540
mmu-miR-139-5p	0.04861	1.473±0.220
mmu-miR-1907	0.01444	1.456±0.302
mmu-miR-30d*	0.03344	1.443±0.404
mmu-miR-367*	0.04441	1.427±0.318
mmu-miR-1927	0.00348	1.418±0.067
mmu-miR-152	0.03278	1.409±0.190
mmu-miR-5097	0.04393	1.404±0.145
mmu-miR-669d-2*	0.01348	1.381±0.178
mmu-miR-669a-5p, -669p	0.03866	1.364±0.338
mmu-miR-93	0.02876	1.349±0.080
mmu-miR-18a	0.02997	1.340±0.196
mmu-miR-30a*	0.04034	1.273±0.171

Downregulated microRNA	P-value	Fold (MCD/control) mean ± SD
mmu-miR-326*	0.00020	0.263±0.038
mmu-miR-2861	0.00046	0.263±0.046
mmu-miR-2137	0.00032	0.274±0.029
mmu-miR-193	0.00009	0.275±0.055
mmu-miR-711	0.00007	0.289±0.069
mmu-miR-1893	0.00221	0.371±0.036
mmu-miR-363-5p	0.00128	0.373±0.159
mmu-miR-3077*	0.00101	0.379±0.042
mmu-miR-211*	0.00315	0.390±0.065
mmu-miR-101b^a^	0.00020	0.418±0.051
mmu-miR-122^a^	0.00031	0.439±0.031
mmu-miR-762	0.01481	0.501±0.135
mmu-miR-5126	0.00178	0.502±0.046
mmu-miR-193b	0.00074	0.527±0.115
mmu-miR-5130	0.02714	0.533±0.186
mmu-miR-328*	0.00114	0.534±0.076
mmu-miR-3960	0.01064	0.541±0.066
mmu-miR-203	0.01068	0.546±0.148
mmu-miR-192	0.03496	0.550±0.203
mmu-miR-92b*	0.02031	0.551±0.186
mcmv-miR-M23-2	0.01668	0.565±0.336
mmu-let-7d*	0.01249	0.569±0.106
mmu-miR-744	0.04968	0.572±0.210
mmu-miR-194^a^	0.00228	0.574±0.078
mmu-miR-5128	0.02948	0.574±0.226
mmu-miR-92a-2*	0.00074	0.577±0.056
mmu-miR-680	0.01109	0.580±0.130
mmu-miR-5122	0.03256	0.585±0.146
mmu-miR-455	0.01402	0589±0.161
mmu-miR-378	0.00397	0.590±0.050
mmu-miR-5132	0.00272	0.597±0.129
mmu-miR-192*	0.01510	0.597±0.127
mmu-miR-3090*	0.04577	0.600±0.179
mmu-miR-5105	0.00690	0.606±0.059
mmu-miR-122*	0.01276	0.613±0.114
mmu-miR-365	0.01093	0.618±0.140
mmu-miR-5115	0.00646	0.639±0.093
mmu-miR-1964-5p	0.02701	0.641±0.395
mmu-miR-3058*	0.01002	0.643±0.177
mmu-miR-3062*	0.01120	0.650±0.184
mmu-miR-486*	0.02615	0.654±0.160
mmu-miR-148a	0.04264	0.662±0.120
mmu-miR-128-2*	0.00337	0.673±0.056
mmu-miR-1934*	0.01077	0.690±0.163
mmu-miR-149*	0.03050	0.698±0.053
mmu-miR-3102	0.04832	0.705±0.226
mmu-miR-3963	0.02807	0.721±0.141
mmu-miR-144*	0.04255	0.722±0.100
mmu-miR-466i-5p	0.00734	0.730±0.063
mmu-miR-30e	0.03175	0.759±0.210
mmu-miR-345-5p	0.01213	0.761±0.136
mmu-miR-721	0.04609	0.761±0.202
mmu-miR-770-3p	0.00998	0.764±0.151
mmu-miR-361*	0.03287	0.771±0.105
mmu-miR-207	0.02826	0.782±0.166
mmu-miR-669c*	0.01509	0.785±0.121
mmu-miR-1306-5p	0.03743	0.796±0.153
mmu-miR-705^a^	0.04984	0.803±0.071
mmu-miR-574-5p	0.03902	0.812±0.168
mmu-miR-3099*	0.03862	0.866±0.111

B, Statistical analysis of miRNAs in liver tissues between MCD-fed mice treated and untreated with metformin		
Upregulated microRNA	P-value	Fold (MCD+metformin/MCD mean ± SD
mmu-miR-879	0.00305	2.003±0.478
mmu-miR-5110	0.02983	1.893±0.673
mmu-miR-466o-3p	0.01079	1.864±0.615
mmu-miR-187*	0.00583	1.792±0.626
mmu-miR-25*	0.01722	1.708±0.589
mmu-miR-676	0.04309	1.633±0.737
mmu-miR-193*	0.03590	1.585±0.344
mmu-miR-335-5p	0.00405	1.550±0.209
microRNA	P-value	mean ± SD
mmu-miR-183	0.04925	1.531±0.522
mmu-miR-1946b	0.01225	1.519±0.334
mmu-miR-380-3p	0.04656	1.492±0.511
mmu-miR-705^a^	0.04487	1.475±0.366
mmu-miR-190b*	0.04529	1.453±0.181
mmu-miR-1187	0.03109	1.436±0.419
mmu-miR-1929	0.03159	1.422±0.441
mmu-miR-194^a^	0.01756	1.406±0.337
mmu-miR-5131	0.03440	1.400±0.356
mmu-miR-532-5p	0.02742	1.398±0.319
mmu-miR-296-5p	0.02568	1.380±0.313
mmu-miR-292-5p	0.01003	1.374±0.192
mmu-miR-1249	0.02044	1.310±0.215
mmu-miR-122^a^	0.00370	1.294±0.169
mmu-miR-101b^a^	0.03923	1.199±0.165

Downregulated microRNA	P-value	Fold (MCD+metformin/MCD) mean ± SD
mmu-miR-376a^a^	0.02593	0.398±0.419
mmu-miR-337-3p	0.00407	0.487±0.206
mmu-miR-301b	0.00559	0.500±0.118
mmu-miR-379	0.04427	0.516±0.453
mmu-miR-410	0.02336	0.528±0.285
mmu-miR-434-5p	0.04516	0.556±0.176
mmu-miR-541	0.04030	0.593±0.236
mmu-miR-21	0.01198	0.595±0.181
mmu-miR-3067*	0.00460	0.596±0.127
mmu-miR-127^a^	0.04540	0.602±0.391
mmu-miR-342-3p^a^	0.00438	0.608±0.175
mmu-miR-300^a^	0.01321	0.608±0.211
mmu-miR-137*	0.02554	0.636±0.163
mmu-miR-470	0.02195	0.637±0.267
mmu-miR-544-3p	0.02228	0.643±0.076
mmu-let-7g*	0.03398	0.653±0.240
mmu-miR-34a^a^	0.04413	0.657±0.294
mmu-miR-181b-1*	0.00829	0.664±0.087
mmu-miR-425	0.03213	0.688±0.209
mmu-miR-1b-5p	0.03025	0.728±0.213
mmu-miR-324-5p	0.04661	0.737±0.187
mmu-miR-221*	0.01899	0.755±0.154
mmu-miR-487b	0.03325	0.791±0.116
mmu-miR-1968	0.01716	0.798±0.085
mmu-miR-320*	0.01552	0.839±0.108

a miRNA expression A is contrary to that in B.

## Abbreviations

miRNA: microRNA
NASH: non-alcoholic steatohepatitis
NAFLD: non-alcoholic fatty liver disease
HCC: hepatocellular carcinoma
TNFα: tissue necrotic factor α
IL-10: interleukin-10
ALT: alanine aminotransferase
AST: aspartate aminotransferase
ALP: alkaline phosphatase
MCD: methionine-and choline-deficient diet
